# Agreement and concurrent validity between telehealth and in-person diagnosis of musculoskeletal conditions: a systematic review

**DOI:** 10.1186/s12998-024-00542-3

**Published:** 2024-06-13

**Authors:** David Oh, Daphne To, Melissa Corso, Kent Murnaghan, Hainan Yu, Carol Cancelliere

**Affiliations:** 1 Canadian Memorial Chiropractic College, 6100 Leslie Street, Toronto, ON M2H 3J1 Canada; 2 Faculty of Health Sciences, Institute for Disability and Rehabilitation Research, Ontario Tech University and Canadian Memorial Chiropractic College, Toronto, ON Canada; 3 Institute for Disability and Rehabilitation Research and Faculty of Health Sciences, Ontario Tech University, Oshawa, Canada

**Keywords:** Telehealth, Diagnosis, Assessment, Virtual care, Musculoskeletal conditions

## Abstract

**Objectives:**

To assess the concurrent validity and inter-rater agreement of the diagnosis of musculoskeletal (MSK) conditions using synchronous telehealth compared to standard in-person clinical diagnosis.

**Methods:**

We searched five electronic databases for cross-sectional studies published in English in peer-reviewed journals from inception to 28 September 2023. We included studies of participants presenting to a healthcare provider with an undiagnosed MSK complaint. Eligible studies were critically appraised using the QUADAS-2 and QAREL criteria. Studies rated as overall low risk of bias were synthesized descriptively following best-evidence synthesis principles.

**Results:**

We retrieved 6835 records and 16 full-text articles. Nine studies and 321 patients were included. Participants had MSK conditions involving the shoulder, elbow, low back, knee, lower limb, ankle, and multiple conditions. Comparing telehealth versus in-person clinical assessments, inter-rater agreement ranged from 40.7% agreement for people with shoulder pain to 100% agreement for people with lower limb MSK disorders. Concurrent validity ranged from 36% agreement for people with elbow pain to 95.1% agreement for people with lower limb MSK conditions.

**Discussion:**

In cases when access to in-person care is constrained, our study implies that telehealth might be a feasible approach for the diagnosis of MSK conditions. These conclusions are based on small cross-sectional studies carried out by similar research teams with similar participant demographics. Additional research is required to improve the diagnostic precision of telehealth evaluations across a larger range of patient groups, MSK conditions, and diagnostic accuracy statistics.

**Supplementary Information:**

The online version contains supplementary material available at 10.1186/s12998-024-00542-3.

## Introduction

Musculoskeletal (MSK) conditions including low back pain, osteoarthritis, and neck pain are the leading cause of disability globally [1]. The 2016 Global Burden of Disease study [2] estimated that one in three people worldwide are living with a painful MSK condition. These conditions bring a high societal burden and contribute significantly to direct (e.g., healthcare) and indirect (e.g., time off work) costs for patients [1, 2]. People with MSK conditions often seek care from a variety of healthcare providers including physiotherapists and chiropractors [3]. However, the COVID-19 pandemic has caused a disruption in normal clinical practice resulting in cancellations of non-urgent and elective surgical procedures and traditional in-person care [4–7]. This has posed a significant problem for people with MSK conditions who need access to healthcare [4–7]. These challenges have caused a need for decision makers, researchers, and clinicians to re-examine the traditional model of healthcare delivery and explore the widespread use of telehealth for the assessment and management of people with MSK conditions [6–8].

Telehealth is defined as ‘the use of telecommunications for medical diagnoses and patient care at a distance’ [9]. One medium for telehealth is the use of web or application-based video and/or audio-conferencing technology for synchronous, or real-time, patient-clinician interactions [9]. The effectiveness of telehealth for clinical interactions has been well studied over several decades and across a wide spectrum of healthcare disciplines [9, 10]. For example, the use of telehealth has been recommended in rural communities where geographical distance to medical specialists is a barrier to patient care [11]. Telehealth may also overcome barriers including ease of access to healthcare providers in areas of provider shortages, cost effectiveness, and decreased patient wait times [12, 13]. Telehealth however, requires both providers and patients to be technologically literate and may pose regulatory barriers, and may not be suitable for peoples with limited access to technological infrastructure (i.e., computers and internet, etc.) and poor communication skills [12, 13]. Telehealth has been reportedly used in MSK practices in the United States, Department of Veterans Affairs for patients with limited access to MSK healthcare providers and also in the United Kingdom to triage patients via telephone consultations [11]. However, the widespread adoption of telehealth use in MSK healthcare disciplines has been slow [11, 12]. One key barrier identified in MSK healthcare is the inability to perform a ‘hands-on’ assessment or treatment with telehealth including neurological tests, palpation, and manual care [4, 13]. The challenges of a ‘hands-off’ approach includes meeting patient expectations of direct interventions through touch but also other contextual factors to the clinical interaction including the atmosphere around the clinical interaction [4]. This can lead to the perception of the clinical encounter being impersonal and potentially less effective compared to standard care [4].

Two recent systematic reviews summarized the validity and reliability findings of individual physical examination components in a clinical assessment for MSK conditions using telehealth [14, 15]. For instance, both reviews reported similar results with low validity and reliability for lumbar spine postural assessments, special orthopaedic tests for the elbow, shoulder, and ankle, and scar assessments with telemedicine [14, 15]. These reviews were limited in scope by exploring the validity and reliability of components of the physical examination. Previous literature has reported the limited validity and reliability for physical examination tests alone to diagnose MSK conditions for the low back and neck [16–18]. It is therefore important to evaluate concurrent validity and inter-rater agreement of telehealth assessment, including all aspects of the clinical assessment (i.e., a detailed health history and physical examination) to reflect an in-person practice model [19–21]. Furthermore, since the onset of COVID-19, a number of studies have been published related to telehealth and MSK care; these should be synthesized [6]. The objective of this systematic review is to systematically search, critically appraise, and synthesize the literature on the concurrent validity and inter-rater agreement of the clinical assessment (history and physical examination) and diagnosis of MSK conditions using synchronous telehealth compared to the standard in-person clinical assessment.

## Methods

### Eligibility criteria

#### Population

This systematic review targeted studies of individuals of all ages who presented to a healthcare provider in a clinic for the clinical assessment or diagnosis of a MSK condition. The International Classification of Diseases lists MSK conditions as a diverse group of over 150 diagnoses that affects the locomotor system: specifically, muscles, bones, joints, tendons, and ligaments [2]. MSK conditions are commonly described by their anatomical location and through their association with pain and impaired physical function despite their variability of pathophysiology [2]. Some examples of commonly studied MSK conditions included in this systematic review include low back and neck pain, osteoarthritis, musculoskeletal injury sequelae, and fractures [1, 2]. Individuals with neurological conditions such as traumatic brain injury, spinal cord injuries, headaches (e.g., migraine, tension-type, cluster, cervicogenic, etc.), and movement disorders (e.g., Parkinson’s Disease, multiple sclerosis, muscular dystrophy, cerebral palsy, amyotrophic lateral sclerosis, etc.) were excluded. Individuals with autoimmune conditions including rheumatoid arthritis and axial spondyloarthropathy were also excluded.

#### Exposure

The exposure is the clinical assessment and diagnosis of MSK conditions by synchronous, or real-time, telehealth using video and audio technologies delivered by any healthcare provider. A clinical assessment includes the combination of a detailed patient history and physical examination (for example, to assess risk factors for serious pathology, characteristics of pain and level of function, onset, barriers to recovery) to establish a correct clinical diagnosis [19, 21]. Studies assessing only single components of a clinical assessment (e.g., range of motion, strength, visual inspection, orthopedic or functional tests, etc.) were excluded. Studies of clinical assessments or diagnoses using asynchronous telehealth (e.g., email, text messages) were excluded.

#### Comparator

The comparator is the standard in-person clinical assessment, including a health history and physical examination of individuals with MSK conditions by any healthcare provider.

#### Outcome

The outcomes are the inter-rater agreement and concurrent validity of clinical diagnoses of individuals with MSK complaints. Inter-rater agreement is defined as the extent to which the responses of two (or more) independent raters are concordant (e.g., percent agreement) [21]. We also examined studies assessing inter-rater reliability which is defined as the degree of agreement between two or more examiners who make independent ratings about the features of a set of subjects (e.g., Cohen’s kappa) [22]. Concurrent validity is defined as a measure of agreement between a particular test and a reference standard [22].

#### Study designs/characteristics

Eligible studies included cross-sectional studies published in English in peer-reviewed journals. The following were excluded: randomized controlled trials, cohort, case reports, case series, case–control, qualitative studies, non-systematic and systematic reviews, clinical practice guidelines, biomechanical studies, laboratory studies, studies not reporting on methodology, unpublished manuscripts, letters, guidelines, commentaries, conference proceedings, editorials, theses, pilot and/or feasibility studies, books, meeting abstracts, lectures, consensus development statements and other descriptive publications.

#### Information sources and search strategy

Five electronic databases (MEDLINE, CINAHL, EMBASE, PsycINFO, and SPORTDiscus) were searched from inception to September 28, 2023. The search strategy was developed following consultation with an experienced health sciences librarian and was reviewed by a second librarian using the Peer Review of Electronic Search Strategies Checklist [23, 24]. The search strategy was developed in MEDLINE (Appendix 1) and adapted to the other bibliographic databases. Search terms included subject headings (e.g., MeSH in MEDLINE) for each database and free text words for the key concepts of telehealth, clinical assessment, diagnosis, in-person assessment, validity, and agreement. EndNote X9 (Clarivate Analytics, Philadelphia, USA) was used as an electronic reference manager to record the number of duplicates identified and delete duplicate references across databases.

#### Data collection and analysis

### Study selection

A two-phase (titles and abstracts; full-text articles) screening process was used to select eligible studies. In phase I screening, pairs of independent reviewers screened citation titles and abstracts to determine the eligibility of studies (categorizing studies as possibly relevant or irrelevant). Pairs of independent reviewers screened possibly relevant studies in full text during phase II screening to determine eligibility and document reasons for exclusion. Reviewers met to discuss disagreements and reach consensus on study eligibility. A third reviewer was consulted in situations where consensus was not reached. Study authors were contacted for additional information as needed when screening, assessing risk of bias, and conducting data extraction.

### Data items and data collection process

The lead author extracted data from eligible studies to build evidence tables. A second reviewer independently extracted study results (e.g., agreement, reliability and validity measures, 95% CI) and any disagreements were discussed to reach consensus. For all other data items, a second reviewer verified by checking the extracted data to minimize error. We used the Landis and Koch [25] reporting guidelines to interpret the strength of reliability. For percentage agreement, poor was defined as 0–0.20, fair as 0.21–0.40, moderate as 0.41–0.60, substantial as 0.61–0.80, and almost perfect as 0.81–1.00 [25].

From each study, extracted data included author, publication year, clinical setting, participant characteristics (e.g., sample size, mean age, sex/gender, and MSK condition), definition of exposure (characteristics of the clinical assessment delivered through telehealth), validity (index test, reference standard, and percent agreement) and/or reliability (intraclass correlation efficient, percent agreement) outcomes, and 95% confidence intervals (CI).

### Risk of bias assessment

Pairs of trained reviewers critically appraised eligible studies using the Quality Appraisal Tool for studies of diagnostic reliability (QAREL) [26] or the Quality Assessment of Diagnostic Accuracy Studies-2 (QUADAS-2) [27]. Reviewers met to reach consensus and a third independent reviewer was consulted to resolve any disagreements.

### Data synthesis

We were guided by synthesis without meta-analysis (SWiM) reporting guidelines to narratively synthesize the data from the low risk of bias studies [28, 29]. This type of synthesis was selected due to the clinical heterogeneity of studies [28]. We used data in the evidence tables to create summary statements. We stratified our synthesis by body region (e.g., hip, knee, shoulder) and type of evidence (agreement, reliability and/or validity).

### Study design

This systematic review was organized and reported based on the Preferred Reporting Items for Systematic Reviews and Meta-Analyses (PRISMA) statement [30].This review protocol was registered with the Open Science Framework on May 10, 2021 (Registration 10.17605/OSF.IO/KVXB2).

## Results

**Fig. 1:**
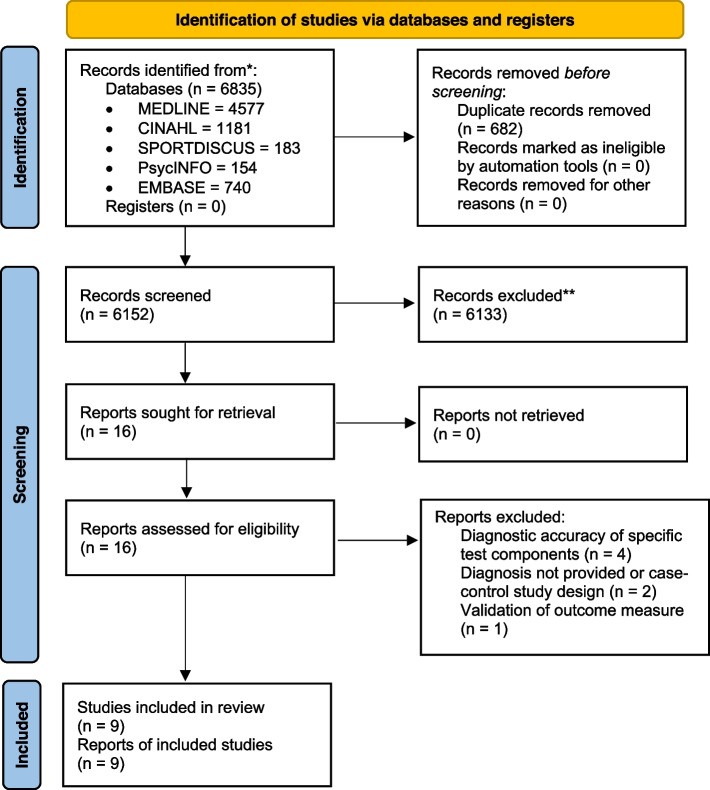
Flow chart of included studies (PRISMA [2020])

We identified 6152 records and retrieved 16 full-text articles, of which 9 studies were eligible and critically appraised (Fig. 1). The primary reasons for exclusion included articles investigating diagnostic accuracy of test components or no diagnosis provided. Of these, eight were rated as overall low risk of bias and one was rated as high risk of bias. No non-English articles were identified. No authors were contacted for additional information.

The nine studies with 321 participants included cross-sectional studies that assessed adults only with a sample size ranging from 11–126, mean age ranging from 23–57.7 years, and percent females ranged from 10–73% [31–39]. Four studies included patients that presented to a tertiary outpatient MSK sports injury clinic [34–37], one study included patients presenting in a university outpatient physiotherapy clinic [32], one study included patients who presented to an outpatient shoulder clinic [31, 38], one study included patients that were referred into an advanced-practice physiotherapy program [33], and one study included patients who were referred to an orthopaedic shoulder clinic [39] (Table 1). Three studies included patients presenting with shoulder pain [31, 32, 39], one study examined patients presenting with low back, knee, or shoulder pain [33], and single studies included patients presenting with elbow, knee, lower limb, low back, and ankle pain respectively [34–38]. Study examiners ranged from final year honours physiotherapy students [32, 34, 37], physiotherapists [35, 36], post-graduate qualified Musculoskeletal Physiotherapists employed in an advanced-practice role [33], and board-certified and fellowship-trained orthopaedic surgeons and orthopaedic residents [31, 38, 39]. All studies used both video and audio technologies for their synchronous telehealth assessments.

**Table 1:** Table of included studies (*n* = 9 cross-sectional studies)

Study, clinical setting, examiner	Participants				Clinical Assessment		Inter-rater Agreement	Concurrent Validity
	Condition/ body region and eligibility	Sample size (unit of analysis)	% Female	Mean age ± SD (years)	Telehealth components	In-person exam components (reference standard)	Inter-rater (% agreement)	% Agreement
Steele et al*.* 2012 [32] Outpatient university-based physical therapy clinic Final year physiotherapy honours students	Shoulder Inclusion: adults (> 18 years old), English speaking, adequate level of cognition and communication Exclusion: poor vision/hearing, concomitant medical conditions (unsafe virtual exam)	22 participants (with 28 reports of shoulder pain)	27.2	30.7 ± 14.2	• Patient interview • Postural analysis • Range of motion (shoulder and adjacent joints) • Self-static muscle tests • Self-applied orthopaedic tests • Self-applied neural tests	• Patient interview • Postural analysis • Joint palpation • Range of motion (shoulder and adjacent joints) • Static muscle tests • Clinician-applied orthopaedic tests • Clinician-applied neural tests	**Primary clinical diagnosis**^**a**^	
							40.74 (same)^c^ 59.26 (similar)^d^	18.52 (same)^c^ 40.74 (similar)^d^
							**Systems diagnosis**^**b**^	
							82.1	78.6
Lade et al*.* 2012 [34] Physiotherapy clinic Final year physiotherapy honours students	Elbow Inclusion: current elbow pain/dysfunction, adults (> 18 years old), English speaking, sufficient cognition/communication Exclusion: poor vision/hearing (prevent from completing exam)	11	10	38 ± 13	• Patient interview • Guided observations • Self-palpation • Range of motion tests with self-applied over pressure • Self-resisted static muscle tests • Self-applied orthopaedic tests • Self-applied upper limb neural tension tests	• Patient interview • Observation • Palpation • Postural analysis • Shoulder and neck screening tests • Joint range of motion tests • Static muscle tests • Clinician-applied orthopaedic tests • Clinician-applied upper limb neural tension tests	**Primary clinical diagnosis**^**a**^	
							73	36
							**Systems diagnosis**^**b**^	
							82	73
Russell et al*.* 2010 [36] Physiotherapy clinic Final year physiotherapy honours students	Ankle Inclusion: current ankle pain/dysfunction, adults (> 18 years old), English speaking, adequate level of cognition and communication Exclusion: concomitant medical condition (unsafe to participate)	15	66.7	24.5 ± 10.8	• Patient interview • Joint range of motion • Modified self-performed functional, movement, or orthopaedic, and neural tests • Posture and gait analysis • Self-palpation • Self-applied static muscle tests	• Patient interview • Postural assessment • Palpation of painful area • Range of motion • Clinician-applied muscle, ligament, joint, and neural tissue tests	**Primary clinical diagnosis**^**a**^	
							93.3 (same)^c^ 6.7(similar)^d^	53.3(same)^c^ 40.0(similar)^d^
							**Systems diagnosis**^**b**^	
							93.3	80.0
Russel et al. 2010 [37] Physiotherapy clinic Physical therapist (experience not reported)	Lower limb musculoskeletal disorders Inclusion: lower limb pain not associated with a joint (clinician opinion), adults (> 18 years old), adequate level of cognition and communication Exclusion: concomitant medical condition (unsafe to participate),	19	73.6	26 ± 13	• Patient interview • Postural assessment • Gait analysis • Functional task analysis • Observation and self-palpation • Joint range of motion • Self-applied manual muscle testing • Neural system tests • Self-applied orthopaedic tests	• Patient interview • Postural assessment • Gait analysis • Functional task analysis • Observation and palpation • Joint range of motion • Manual muscle testing • Neural system tests • Orthopaedic tests	**Primary clinical diagnosis**^**a**^	
							84(same)^c^ 100(similar)^d^	68(same)^c^ 79(similar)^d^
							**Systems diagnosis**^**b**^	
							100	95.1 k = 0.98 (95% CI; 0.022–0.048)
Richardson et al*.* 2016 [35] Physiotherapy clinic Physiotherapist (experience not reported)	Knee Inclusion: Adults (> 18 years old), with symptoms of knee pain, adequate level of cognition and communication, ability to independently mobilize Exclusion: concomitant medical conditions that would prevent them from safely completing a physical exam	18	55.6	23 ± 7	• Patient interview • Postural examination • Gait analysis • Active movement • Self-palpation • Self-resisted muscle testing • Self-applied orthopaedic tests • Self-applied neurological and neurodynamic movements	• Patient interview • Postural examination/observation • Palpation • Joint range of motion • Manual muscle tests • Orthopaedic tests • Neurological and neurodynamic testing • Gait and functional task analysis	**Primary clinical diagnosis**^**a**^	
							89(same)^c^ 100 (similar)^d^	67(same)^c^ 89(similar)^d^
							**Systems diagnosis**^**b**^	
							94	94
Cottrell et al*.* 2018 [33] Advanced practice physiotherapy screening clinic Post-graduate qualified Musculoskeletal Physiotherapists employed in an advance-practice role (2–9 years of experience)	Chronic lumbar spine, knee, or shoulder conditions Inclusion: adults (> 18 years old) with non-urgent or routine assessments for conditions in the lumbar spine, knee, or shoulder, available relevant radiological investigations < 12 months Exclusion: medical conditions that may preclude a safe examination, hearing or visual impairment, inability to mobilize independently, needed a translator	**Total**			• Patient interview • Standard physical exam procedures with modification (self-applied pressure, self-applied orthopaedic tests)	• Patient interview • Physical examination (pragmatic and remained unchanged from standard clinical practice)	**Total**	N/A
		42	57.1	52.7 ± 14.5			38.1 (same)^c^ 45.2 (similar)^d^ 16.7 (different)^e^	
		**Lumbar spine**					**Lumbar spine**	
		14	57	51.6 ± 13.5			42.9 (same)^c^ 50 (similar)^d^ 7.1 (different)^e^	
		**Knee**					**Knee**	
		14	43	48.9 ± 14.2			42.9 (same)^c^ 35.7 (similar)^d^ 21.4 (different)^e^	
		**Shoulder**					**Shoulder**	
		14	71	57.7 ± 15.5			28.6 (same)^c^ 50 (similar)^d^ 21.4 (different)^e^	
Rabin et al*.* 2022 [31] Outpatient shoulder clinic Board-certified and fellowship-trained orthopaedic surgeons	Inclusion: adults (> 18 years old), unilateral shoulder complaint, access to cellular phone with video call application Exclusion: lack of good communication skills	47	36	44.6 ± 22.0	• History (over cell phone video call) • Modified physical examination (visual examination and self-assisted or self-applied tests including range of motion, manual muscle testing, palpation and special tests)	• Demographic information • History • Physical examination • Review of pertinent imaging as per usual practice	85.1% agreement k = 0.82 (95% CI; 0.69–0.94)	N/A
Wang et al*.* 2022 [39] Orthopaedic shoulder clinic Shoulder surgeon and two independent observers	Inclusion: new patients aged ≥ 18 years presenting with unilateral shoulder complaints Exclusion: patients with bilateral shoulder complaints, acute fracture, or infection and non–English language speakers	32	47	50.2 ± 16.2	• Blinded history and third-party descriptions of patient imaging • Modified shoulder examination maneuvers – battery of 40 tests categorized based on motion type, direction, and symptoms (coached by a non-treating team member)	• Blinded history and third-party descriptions of patient imaging • Standard battery of 40 tests categorized based on motion type, motion direction, and symptom type	Instability KR-20 = 0.56 SLAP/biceps KR-20 = 0.59 Arthritis KR-20 = 1.00 Rotator cuff KR-20 = 0.85 Acromioclavicular joint KR-20 = 0.00 Cervical KR-20 = 1.00 Frozen shoulder KR-20 = 0.93 Other KR-20 = 0.88	N/A
Moreira Dias Jr et al*.* 2023 Orthopaedic outpatient 17 medical professionals with 3 different degrees of expertise (orthopaedic residents, orthopaedists who were not specialists in spine surgery, orthopaedists who specialized in spine surgery)	Inclusion: adults with low back pain lasting 6 weeks or more who were awaiting an appointment for their first orthopedic care visit at a local referral center Exclusion: individuals with suspected emergency situations, symptoms other than low back pain, or those without digital resources	126	66.4	47.8 ± 13.1	• Digital questionnaire completed by the participants • Adapted physical examination	• Digital questionnaire completed by the participants • Traditional physical examination	79.5% agreement k = 0.585, P < 0.001	N/A

*N/A* Not applicable, *k* Cohen’s kappa, *KR-20* Kuder-Richardson formula 20

^a^Primary clinical diagnosis is defined as an exact pathoanatomical structure or condition that the participant presented with [33]

^b^Systems diagnosis is defined as the anatomical system (e.g., muscle, bone, articular, neural, etc.) that contained the primary pathology [33]

^c^Exact/same is defined as an exact match including minor variations in diagnostic labelling [33]

^d^Similar is defined as a significant overlap in structure or source of symptoms [33]

^e^Different is defined as large differences in structure or source of symptoms [33]

In six studies [32–37], the telehealth and in-person examiners were asked to diagnose participants following their examination based on an exact primary clinical diagnosis and a systems diagnosis. A primary clinical diagnosis was defined as the exact anatomical structure involved, and a systems diagnosis as a broader category (e.g., muscle, tendon, nerve) that was the cause of the patient’s symptoms [32–37]. Their diagnoses would be compared as same, similar, or different [32–37]. Cottrell et al*.* [33] provided an operational definition that outlined same diagnosis as an exact match including minor variations in diagnostic labelling, similar diagnosis as a significant overlap in structure or source of symptoms, or different as large differences in structure or source of symptoms. A similar definition was provided in five other studies [32, 34–37]. Two studies asked examiners to classify a shoulder diagnosis based on distinct subgroups [31, 39]. Moreira Dias Jr et al*.* [38] asked examiners to determine a low back pain diagnosis based on the International Classification of Diseases, 10th Revision [40].

Studies did not provide sensitivity and specificity because there are multiple diagnoses in each study. For example, in Steele et al*.* study [32], participants with shoulder pain were diagnosed based on a pathoanatomical structure, condition or movement dysfunction (e.g., supraspinatus tendinitis and functional subacromial impingement with neural tightness and mechanosensitivity, chronic acromioclavicular joint pain due to degeneration, or mild glenohumeral joint laxity and rotator insufficiency). Therefore, statistical analysis included percentage agreements, Cohen’s kappa, and Kuher-Richardson formula 20 (KR-20) are provided.

Nine studies assessed inter-rater agreement of the telehealth examination (Table 1) [31–39]. Percent agreement between same primary clinical diagnoses was substantial to almost perfect, ranging from 73–93.3% (Table 1) [34, 36]. For systems diagnosis, inter-rater agreement was almost perfect ranging from 82% in the elbow [34] to 100% in the lower limb [36]. Four studies investigated agreement of the shoulder [31–33, 39]. The highest agreement for same primary clinical diagnosis of 85.1% was reported by Rabin et al*.* [31], followed by 40.7% [32], and 28.6% agreement [33]. Wang et al*.* [39] calculated KR-20 reliability scores which ranged from 1.00 for shoulder arthritis and shoulder complaints of cervical origin to 0.00 for acromioclavicular joint-related shoulder pain. Two studies that investigated agreement for knee conditions and reported same primary clinical diagnoses of 89% [35] and 42.9% [33]. By individual body region, the highest agreement for primary clinical diagnosis was reported in the ankle (93.3%) [37], lower limb (84%) [36], low back (79.5%) [38], elbow (73%) [34], and low back (42.9%) [33]. Inter-rater agreement was generally much higher for same plus similar primary clinical diagnoses (Table 1).

Five studies assessed concurrent validity with no studies examining the same body region [32, 34–37]. Percent agreement between same primary clinical diagnosis was poor to substantial, ranging from 18.5–67% (Table 1) [32, 35]. Validity was graded moderate to almost perfect for same plus similar primary clinical diagnoses. For systems diagnosis, validity was substantial to almost perfect, ranging from 73% in the elbow [34] to 95.1% in the lower limb [36]. Highest agreement for systems diagnosis was reported in the lower limb (95.1% agreement), then the knee (94%), ankle (80%), elbow (73%), and shoulder (78.6%) [32, 34–37].

Eight of the included studies were graded as low risk of bias based on the QAREL checklist [31–37, 39] and one was graded as high risk of bias [38] (Table 2). Five of the validity studies were graded as low risk of bias based on the QUADAS-2 checklist [32, 34–37] (Table 3). The studies had a wide variability of sampling strategies and blinding protocols. For example, some studies reported convenience sampling [33], consecutive sampling [31, 32, 34, 36, 37], and sequential sampling [35]. Two studies [38, 39] did not report any sampling strategy. Five studies reported pilot testing with two or four subjects to familiarize the examiners with the virtual testing procedures [32, 33, 35–37]. Six studies reported blinding protocols between the examiners and those involved in data analysis [32–37]. Seven studies reported the specific telehealth technology used [32–38]. Rabin et al*. *[31] did not report blinding between examiners and reported that a cell phone with a video call application was used. Additionally, Wang et al*.* [39] did not report which telehealth technology was used. Furthermore, examiner experience and discipline varied between the studies. Three studies [32, 34, 37] included final year honours physiotherapy students, two studies included physiotherapists without mention of years of clinical experience [35, 36], advanced-practice physiotherapists working in a Neurosurgical & Orthopaedic Physiotherapy Screening Clinic and Multidisciplinary Service [33], orthopaedic residents, orthopedists who specialized in spine surgery, and other orthopaedics [38], and board-certified and fellowship-trained orthopaedic surgeons [31]. It was unclear as to the treating physician’s experience and discipline in the Wang et al*. *study [39] and who was performing the evaluation.

**Table 2:** Summary of assessment of risk of bias for accepted studies based on the modified quality appraisal tool for studies of diagnostic reliability (QAREL) criteria for diagnostic reliability studies

References	Clear study objective	Representative sample	Representative raters	Inter-rater blinding	Intra-rater blinding	Raters blinded to reference standard	Raters blinded to other clinical info	Raters blinded to other cues	Varied order of exam	Appropriate time between measurements	Test applied appropriately	Appropriate statistical measures
Richardson et al*.* 2016	Y	Y	Y	Y	N/A	N/A	UC	N/A	Y	Y	Y	Y
Lade et al*.* 2012 [34]	Y	Y	Y	Y	N/A	N/A	UC	N/A	Y	Y	Y	Y
Steele et al*.* 2012 [32]	Y	Y	Y	Y	N/A	N/A	UC	N/A	Y	Y	Y	Y
Russell et al*.* 2010^a ^[36]	Y	Y	Y	Y	N/A	N/A	UC	N/A	Y	Y	Y	Y
Russell et al*.* 2010^b^ [37]	Y	Y	Y	Y	N/A	N/A	UC	N/A	Y	Y	Y	Y
Rabin et al*.* 2022 [31]	Y	Y	Y	UC	N/A	N/A	UC	N/A	N	Y	Y	Y
Cottrell et al*.* 2018 [33]	Y	Y	Y	Y	N/A	N/A	Y	N/A	Y	Y	Y	Y
Moreira Dias Jr et al*.* 2023	Y	Y	Y	Y	N/A	N/A	UC	N/A	UC	UC	UC	Y
Wang et al*.* 2022 [39]	Y	Y	Y	Y	N/A	N/A	Y	N/A	Y	Y	Y	Y

*Y* Yes, *N* No, *NA* Not Applicable, *UC* Unclear

^a^Telerehabilitation mediated physiotherapy assessment of ankle disorders

^b^The diagnostic accuracy of telerehabilitation for nonarticular lower-limb musculoskeletal disorders

**Table 3:** Summary of assessment of risk of bias for accepted studies based on the modified quality assessment of diagnostic accuracy studies-2 QUADAS-2) criteria for diagnostic accuracy studies

References	Consecutive sample	Case–control design avoided	Avoid inappropriate exclusions	Blinded to ref std results	Prespecified threshold	Described ref std	Appropriate ref std	Blinded to index test results	Appropriate time interval	All patient received ref std	All patients received same ref std	All patients included in analysis
Richardson et al*.* 2016	Y	Y	Y	Y	Y	Y	Y	Y	Y	Y	Y	Y
Lade et al*.* 2012	UC	Y	Y	Y	Y	Y	Y	Y	Y	Y	Y	Y
Steele et al*.* 2012	UC	Y	Y	Y	Y	Y	Y	Y	Y	Y	Y	Y
Russell et al*.* 2010	UC	Y	Y	Y	Y	Y	Y	Y	Y	Y	Y	Y
Russell et al*.* 2010 (1950)	UC	Y	Y	Y	Y	Y	Y	Y	Y	Y	Y	Y

*Y* Yes, *N* No, *NA* Not Applicable, *UC* Unclear

^a^Telerehabilitation mediated physiotherapy assessment of ankle disorders

^b^The diagnostic accuracy of telerehabilitation for nonarticular lower-limb musculoskeletal disorders

In six studies, participants were randomized to receive either telehealth assessment or in-person assessment by a computer-generated randomized block design of four or six [32–37]. One study reported the use of a random number generator [39]. In the study by Rabin et al*. *participants were not randomized to either telehealth or in-person assessments, but the order of examination was scheduled based on examiner availability [31]. Participants were also given the choice to elect for a virtual assessment in an attempt to minimize wait time at the clinic [31]. No randomization was reported by Moreira et al. [38].

In five studies, independent examiners performed physical examinations, but the history component was randomly performed by either the telehealth or in-person examiner while the other examiner was a passive observer [32, 34–37]. This may have led to the introduction of bias in the form of cues. Two studies performed independent history and physical examinations that was at the discretion of each examiner [31, 32]. Lastly, participants were also offered a rest period ranging from 10–30 min in some studies [32, 33, 36, 37]. This brief rest period could have potentially resulted in participants learning movements and tests that may have impacted findings. One study reported that participants filled out a digital questionnaire for the clinical history [38] and another reported that examiners were provided a blinded history with third-party descriptors of patient imaging [39]. In one study, while in-person and telehealth examinations were scheduled for different dates, the time interval was however not specified by Moreira et al. Lastly, while most studies reported that the components of the assessment (history and physical examination) were at the discretion of the in-person or telehealth examiner, one study did not report what components were included in the examination [38] and another reported a standardized shoulder assessment battery of 40 tests [39].

## Discussion

Our review evaluated telehealth assessments in the diagnosis of MSK conditions based on the contributions from a clinical history and physical exam. In adults, we found evidence that telehealth assessments had moderate to almost perfect inter-rater agreement and poor to substantial concurrent validity based on same primary clinical diagnoses of MSK conditions for the low back, knee, shoulder, lower limb, ankle, elbow, and shoulder [31–39]. For systems-based diagnosis, inter-rater agreement was almost perfect and concurrent validity was substantial to almost perfect [32–37]. Percent agreement for both inter-rater agreement and concurrent validity improved when considering same plus similar primary clinical diagnosis [32–37]. Our results are based on small cross-sectional studies that had a similar population, conducted in similar settings, and with the same researchers [32–37]. While the provided agreement percentages and kappa statistics offer valuable insights, a more comprehensive diagnostic accuracy evaluation, including sensitivity, specificity, and predictive values, would enrich our understanding. Furthermore, most of the included studies did not report CI, or had wide CIs which impacted our confidence in interpreting the precision of their results [32–37]. Lastly, the included studies investigated MSK conditions of the low back, knee, shoulder, lower limb, elbow, and ankle. This could limit the generalizability of the review findings.

We believe there are several explanatory factors given the wide range of our results. First, five studies were performed with similar authorship teams, study designs, telehealth technologies, and in populations with a mean age below 50 [32, 34–37]. This could potentially explain why these studies had higher agreement and validity outcomes. Age may have also impacted the type of MSK condition that presented for evaluation. Three studies investigated MSK conditions of the lower extremity [35–37], with two being published in the same year [36, 37]. These three studies reported higher agreement and validity scores compared to the upper extremity MSK conditions. This may reveal that examiners are more congruent with diagnosing conditions of the lower extremity or show a diagnostic challenge for upper extremity conditions. Cottrell et al*.* [33] recruited participants from a specialty referral-only practice with chronic low back, knee, or shoulder conditions. This unique population may have influenced their findings as examiners reported a total diagnostic agreement of 38.1% [33]. While other studies did not report the average onset of symptoms for participants, chronicity of symptoms may have contributed to the diagnostic challenge in this case. Lastly, Rabin et al*.* and Wang et al*.* [31, 39] included participants that presented to an outpatient shoulder clinic for evaluation by an orthopaedic surgeon. This may have influenced the type or severity of condition that presented compared to a community physiotherapy or chiropractic clinic. These studies also asked examiners to categorize patient diagnosis based on predetermined subgroups. Our results showed the highest agreement outcomes using subgroup diagnoses compared to the other studies evaluating patients with shoulder pain [32, 33].

Previous systematic reviews have investigated the validity and reliability of components of the virtual and in-person physical examination. These reviews reported moderate to good inter-rater reliability for range of motion of MSK conditions in the shoulder, low back, and knee through visual inspection or virtual goniometry and lower scores for postural assessment of the low back and self-applied orthopaedic tests for the elbow, shoulder, and ankle [14, 15]. While it is important to understand the clinometric properties of these virtual tests, establishing an accurate clinical diagnosis for MSK conditions should be derived from a comprehensive assessment including the patient history and thorough physical examination [19].

### Strengths and limitations

Our review has strengths. We used a comprehensive, peer-reviewed literature search strategy developed in collaboration with an experienced health sciences librarian [24]. Secondly, we did not rely on summary scores or arbitrary cut-off points during the risk of bias assessments; instead, we based our judgements on critical flaws captured in the QAREL and QUADAS-2 tools [26, 27]. We also attempted to minimize potential bias and inclusion of all relevant studies using a consensus process among reviewers to determine study eligibility and risk of bias.

Our review also has limitations. Some limitations include that our study only identified articles in English as studies in other languages may have reported different results. We also excluded grey literature from our review. This could potentially introduce publication bias as these sources may contain alternative results. Lastly, our results may be impacted as studies with positive or significant results are more likely to be published leading to an overestimation of our results.

### Clinical implications

Clinicians may consider synchronous telehealth as a feasible option based on clinical expertise and patients’ preference and values and decide if further in-person care is necessary. There are several factors to ensure ideal conditions for the telehealth encounter. One of the well documented barriers to virtual care include patients who present in sub-optimal settings with poor lighting, bandwidth limitations, and low camera resolutions [41, 42]. Clinicians must ensure patients are prepared in a location with adequate space and light. Similarly, some patients with low digital health literacy or difficulty with access to appropriate technology may not be suitable candidates for telehealth as they may be at risk of poorer participation and clinical outcomes [41]. Developing good communication skills are foundational to the success of remote healthcare [11, 12, 18, 41–44]. When considering telehealth assessments, clinicians should utilize all available information including videos, still images, self-demonstrations, and verbal cueing and coaching to lead participants through a virtual physical examination [32–39]. Other factors including looking at the camera to simulate direct eye contact and avoiding multitasking during the assessment can help build engagement, rapport, and greater participation from patients [41–43].

Looking ahead, it is imperative for future research to delve into the diagnostic accuracy of telehealth assessments across diverse patient populations and a broader spectrum of MSK conditions. It is recommended that future studies include different patient populations (e.g., adolescents, older adults, etc.) with different MSK conditions, in various settings including primary practice to reflect the heterogeneity of clinical practice. Additionally, expanding investigations to other patient groups, such as those presenting neurological complaints, will ensure our findings are applicable in the varied landscape of clinical practice. Also, larger participant sample sizes and a more diverse assessment of diagnostic accuracy, which takes into account sensitivity, specificity, and predictive values would provide a robust evaluation to draw conclusions from.

## Conclusion

Telehealth diagnoses for specific MSK conditions, including those related to the lower back, shoulder, elbow, lower limb, knee, and ankle, have shown moderate to high concurrent validity and inter-rater agreement. This evidence suggests that telehealth might be a promising alternative to traditional in-person diagnosis. However, it is crucial to recognize that our current understanding is primarily based on small cross-sectional studies that shared similarities in settings, populations, and the research teams behind them.

### Supplementary Information

Supplementary Material 1.

## Data Availability

This review protocol was registered with the Open Science Framework on May 10, 2021 (Registration 10.17605/OSF.IO/KVXB2). Additional data are available from the corresponding author on request.
