# Supplementary material for: Agreement and concurrent validity between telehealth and in-person diagnosis of musculoskeletal conditions: a systematic review
Source: Chiropr Man Therap. 2024 Jun 13;32:21. doi: 10.1186/s12998-024-00542-3 (PMC11177434; doi:10.1186/s12998-024-00542-3)
Supplement: Supplementary file 1 — Supplementary Material 1. [file 12998_2024_542_MOESM1_ESM.docx]

**Appendix.** MEDLINE search strategy

**Dr David Oh - Ovid MEDLINE - Draft Search Strategy APRIL 16, 2021**

1. Telemedicine/ no exp
2. Telerehabilitation/
3. Cell Phone/ no exp
4. Internet/
5. Remote Consultation/ no exp
6. Smartphone/
7. Telecommunications/ no exp
8. Telenursing/
9. Telephone/ no exp
10. Videoconferencing/ no exp
11. cybermed*
12. e-consult* or econsult*
13. eHealth or e-Health
14. internet or online or remote or virtual or video or tele*
15. mHealth or m-Health
16. phone or cell phone or mobile phone or cellular phone or smartphone or iphone
17. real time or real-time
18. telecare or tele-care
19. teleconferenc* or tele-conferenc*
20. teleconsult* or tele-consult*
21. telehealth or tele-health
22. telehomecare or tele-homecare
23. telemat*
24. telemed* or tele-med*
25. telenurs*
26. teletechnol*
27. televideo or tele-video
28. video confer* or videoconfer*
29. web based or web-based
30. (consult* adj3 (digital or distance or distant or electronic or home or mobile)
31. (practice* adj2 (digital or distance or distant or electronic or home or mobile)
32. (care* adj4 (digital or distance or distant or electronic or mobile
33. visit* adj4 (digital or distance or distant or electronic or mobile
34. check-up* adj4 (digital or distance or distant or electronic or mobile
35. (health adj4 (distance or distant
36. (communicat* adj4 (digital or distance or distant or mobile
37. app* adj2 (digital or distance or distant or electronic or home or mobile
38. (assess* adj2 (digital or distance or distant or electronic or home or Internet or mobile or online or remote or virtual or tele* or video*)).ab,ti.
39. (exam* adj2 (digital or distance or distant or electronic or home or Internet or mobile or online or remote or virtual or tele* or video*)).ab,ti.
40. diagnos* adj2 (digital or distance or distant or electronic or home or Internet or mobile or online or remote or virtual or tele* or video*)).ab,ti.
41. **or/1-40 [**Telemedicine]**
42. Physical Examination/ exp
43. Neurological Examination/
44. Medical History Taking/
45. Disability Evaluation/
46. Gait/
47. Palpation/
48. Range of motion, articular/
49. Vital signs/
50. Muscle strength/
51. clinical exam* ab.ti.
52. clinical eval* ab.ti.
53. clinical diagnos*
54. assess* ab.ti.
55. physical exam*.ab.ti.
56. gait
57. palpat*
58. range of motion or range-of-motion
59. goniomet*
60. muscle strength.ab.ti.
61. (history taking or history-taking)
62. interview*
63. medical histor*
64. health histor*
65. family histor*
66. neurological exam*ab.ti.
67. function test
68. neurodynamic test
69. orthopaedic test or orthopedic test
70. **or/42-69 [**Clinical assessment]**
71. in-person
72. face-to-face
73. **or/71-72 [** ]**
74. MH "Reproducibility of Results+"
75. MH "Sensitivity and Specificity"
76. MH "Predictive Value of Tests”
77. MH "Likelihood Functions"
78. MH “False Positive Reactions”
79. MH “False Negative Reactions”
80. MH "ROC Curve"
81. MH "Odds Ratio"
82. MH Observer Variation
83. reliab*
84. reproducibility
85. test-retest*
86. valid*
87. **PT validation study**
88. **or/ 74-87 [** validity/reliability]**
89. **41 AND 70 AND 73 AND 88**
90. LIMIT NOT (animal* NOT human*)
91. LIMIT NOT PT (comment or clinical conference or congress or consensus development conference or editorial or letter or guideline or practice guideline)
